# The Novel CXCL12γ Isoform Encodes an Unstructured Cationic Domain Which Regulates Bioactivity and Interaction with Both Glycosaminoglycans and CXCR4

**DOI:** 10.1371/journal.pone.0001110

**Published:** 2007-10-31

**Authors:** Cédric Laguri, Rabia Sadir, Patricia Rueda, Françoise Baleux, Pierre Gans, Fernando Arenzana-Seisdedos, Hugues Lortat-Jacob

**Affiliations:** 1 Institut de Biologie Structurale (IBS), UMR 5075 CNRS CEA UJF, Grenoble, France; 2 Institut Pasteur, Unité de Pathogénie Virale Moléculaire INSERM 819, Paris, France; 3 Institut Pasteur, Unité de Chimie Organique, URA 2128, Paris, France; University of Cambridge, United Kingdom

## Abstract

**Background:**

CXCL12α, a chemokine that importantly promotes the oriented cell migration and tissue homing of many cell types, regulates key homeostatic functions and pathological processes through interactions with its cognate receptor (CXCR4) and heparan sulfate (HS). The alternative splicing of the *cxcl12* gene generates a recently identified isoform, CXCL12γ, which structure/function relationships remain unexplored. The high occurrence of basic residues that characterize this isoform suggests however that it could feature specific regulation by HS.

**Methodology/Principal Findings:**

Using surface plasmon resonance and NMR spectroscopy, as well as chemically and recombinantly produced chemokines, we show here that CXCL12γ first 68 amino acids adopt a structure closely related to the well described α isoform, followed by an unfolded C-terminal extension of 30 amino acids. Remarkably, 60 % of these residues are either lysine or arginine, and most of them are clustered in typical HS binding sites. This provides the chemokine with the highest affinity for HP ever observed (Kd = 0.9 nM), and ensures a strong retention of the chemokine at the cell surface. This was due to the unique combination of two cooperative binding sites, one strictly required, found in the structured domain of the protein, the other one being the C-terminus which essentially functions by enhancing the half life of the complexes. Importantly, this peculiar C-terminus also regulates the balance between HS and CXCR4 binding, and consequently the biological activity of the chemokine.

**Conclusions/Significance:**

Together these data describe an unusual binding process that gives rise to an unprecedented high affinity between a chemokine and HS. This shows that the γ isoform of CXCL12, which features unique structural and functional properties, is optimized to ensure its strong retention at the cell surface. Thus, depending on the chemokine isoform to which it binds, HS could differentially orchestrate the CXCL12 mediated directional cell kinesis.

## Introduction

CXCL12, also known as SDF-1 (Stromal cell-Derived Factor-1), belongs to the growing family of chemokines, a group comprising some fifty low molecular weight proteins, best known to mediate leukocyte trafficking and activation [1]. CXCL12, initially identified from bone marrow stromal cells and characterized as a pre-B-cell stimulatory factor [2], is constitutively expressed within tissues during organogenesis and adult life [3], [4]. This chemokine, highly conserved among mammalian species, is a key regulator of oriented cell migration. As such, it orchestrates a very large array of functions, both during development and adult life [5]–[9] and is also importantly involved in a number of pathogenic mechanisms [10], [11]. These physiopathological effects are mediated by the G-protein coupled receptor CXCR4, to which the chemokine binds and triggers cell signaling [6], [12]. In addition to these physiological functions, CXCL12 is a potent inhibitor of the cellular entry of CXCR4-dependent human immunodeficiency virus [12]. Recently, we have documented that CXCR7 (RDC-1), also binds to- and is activated by- CXCL12 [13], although the biological role played by this couple remains to be further characterized.

From a structural view point, CXCL12 has a typical chemokine fold stabilized by two disulfide bonds: it consists of a poorly structured N-terminus of 10 residues, followed by a long loop, a 3_10_ helix, a three stranded β-sheet and a C-terminal α-helix. Up to recently, two CXCL12 isoforms, arising from the alternative splicing of a single gene [14] have been studied. The predominant α form encodes a 68 amino acid peptide, while the β one contains four additional amino acids at the C terminus. Most functional data on CXCL12 were obtained from CXCL12α and β, while to date, three isoforms (α, β and γ) and up to six isoforms (α, β, γ, δ, ε and φ) of CXCL12 have been found in rodents [15] and human [16], respectively. All these isoforms share the same three first exons corresponding to the α isoform (residues 1 to 68), but differ in their fourth exon, which gives rise to a specific C-terminal domain for each of them.

It has become clear that biological information required to run the chemokine system is not only stored in the sequences of the proteins involved, but also in the structure of a class of polysaccharide called glycosaminoglycans (GAGs), in particular heparan sulfate (HS), to which most chemokines bind [17] primarily through ionic interactions. Anchored to various core proteins to form proteoglycans, these complex polysaccharides are ubiquitously found on the cell surface and within the extracellular matrix [18]. These molecules have a unique molecular design in which sulfated disaccharide units are clustered in specific domains of variable length and sulfation profile, providing the chain a large array of different protein binding sites [19]. HS are importantly implicated in the regulation of the proteins they bind, and have recently emerged as critical regulators of many events involving cell response to external stimuli. Current models suggested that HS enhance chemokine immobilization and forms haptotactic gradients of the protein along cell surfaces, hence providing directional cues for migrating cells [20], protects chemokines from enzymatic degradation [21], and promotes local high concentrations at the cell surface, facilitating receptor binding and downstream signaling (for review see [22]). *In vivo* data support the view that, within tissues, CXCL12 is sequestered by HS [23].

**Figure 1:**
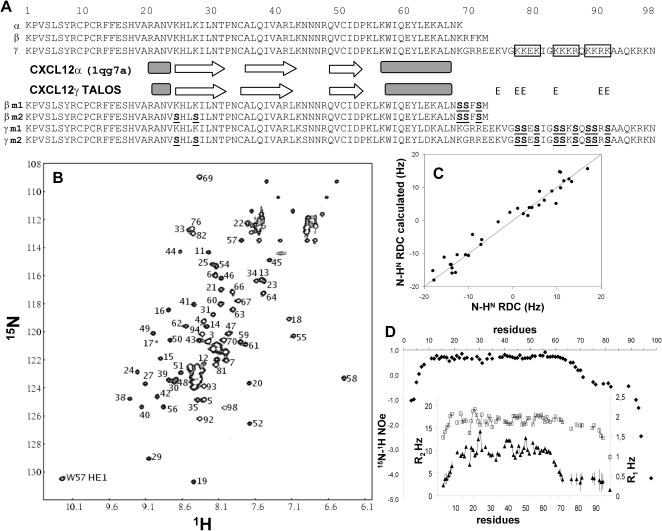
CXCL12γ has an unstructured C-terminal domain but is identical to CXCL12α in the 1-68 region. (A) Sequences of the wild type and mutant CXCL12α, β and γ isoforms produced and used in this study (mutated residues are underlined). The secondary structures of CXCL12γ 1–68 domain and CXCL12α are almost identical (black boxes: α helices, white arrows: β strands, E: extended conformation). (B) ^15^N-HSQC spectrum of CXCL12γ (1 mM, 30°C), on which only non overlapping amide protons were indicated for clarity. Residues from the γ extension are clustered between 8–8.5 ppm ^1^H frequency. (C) CXCL12γ 1–68 domain and CXCL12α fold similarly. A good correlation (Chi^2^ = 74) is observed between N-H^N^ RDCs (CXCL12γ 10–64) with RDCs backcalculated from the CXCL12α structure. (D) ^15^N-^1^H heteronuclear NOes, longitudinal (R1; square) and transversal (R2; triangle) relaxation rates on CXCL12γ. CXCL12γ is folded between residues 10 and 64 and the γ extension is disordered with low NOe, R2 and R1 values.

CXCL12α binding to HS critically involves amino acids K24 and K27, which together with R41 form the essential part of the HS-binding site [24] and are distinct from those required for binding to CXCR4. Given that the minor δ, ε and φ isoforms lack any recognizable HS-binding motif in their carboxy-termini, it can be hypothesized that like CXCL12α, the K24-K27-R41 epitope recapitulates their ability to interact with HS. The situation could be radically different for the novel CXCL12γ isoform. It is indeed characterized by a distinctive 30 amino acids long C-terminal peptide, remarkably conserved between rodents and human, which contains as much as 18 basic residues (B), 9 of which being clustered into three putative BBXB HS-binding domains (Fig. 1A). The existence of carboxy-encoded HS-binding motifs suggests that this isoform could interact with enhanced affinity and/or different selectiveness with GAGs to accomplish specific functions. However, the structure/function relationships of this very peculiar CXCL12 isoform have not been explored yet. Here, we show that CXCL12γ 1 to 68 domain adopts a structure closely related to the α isoform and has an unstructured C-terminal region. This domain reduces CXCR4 occupancy, but in contrast broadens the spectrum of GAG to which the chemokine binds. Moreover, it stabilizes the CXCL12γ/HS complex and, in cooperation with the K24-R41 epitope, provides the chemokine with the highest affinity for GAGs ever observed for any chemokine.

## Results and Discussion

### Wild type and mutants CXCL12 production

The *CXCL12γ* cDNA, obtained from Balb/C mouse brain mRNA was cloned and over expressed in *E. coli*, purified to homogeneity, and characterized by mass spectrometry, NMR and amino acid analysis. The preparation routinely yielded 4–5 mg of purified protein per liter of bacterial culture. Wild type and mutants CXCL12α, β and γ, (Fig. 1A) were also produced by chemical synthesis and characterized by ion spray mass spectrometry and HPLC analysis. Final purity of all samples was found to be, on average, in the range of 90–95%. The biological activity (chemotaxis) of the recombinant chemokine and its chemically synthesized homologue was identical (data not shown).

### CXCL12γ has a typical chemokine fold in the 1–68 domain and an unstructured C-terminal extension

CXCL12α structure has been solved both by X ray crystallography [25], [26] and NMR spectroscopy [27]. The α and β isoform structures are similar [28] but no information has yet been reported for CXCL12γ. To perform structural and binding studies, recombinant CXCL12γ was purified from cells grown in ^15^NH_4_Cl and ^13^C-glucose supplemented medium. Backbone resonances were assigned and the secondary structure content evaluated from ^13^C, ^15^N and ^1^H frequencies (TALOS [29]). The fold similarity of CXCL12γ and α was assessed by recording orientational informations (N-H^N^ Residual Dipolar Couplings (RDC)) of partially aligned molecules in dilute liquid crystal [30], and NMR relaxation experiments were used to evaluate regions of flexibility.

The first 68 residues of CXCL12γ have a spectrum very similar to that of CXCL12α [28], [31], enabling the identification of most residues by visual inspection. This was confirmed by the complete assignment of CXCL12γ residues, but K1, E73 and K84 (Fig. 1B). However, the assignment of CXCL12γ 69–98 remains tentative for the repeated KK motifs which present very similar backbone chemical shifts. Secondary structure prediction from the backbone chemical shifts indicated almost identical secondary structure content for CXCL12α and γ. Forty two N-H^N^ RDCs, in the 10–64 domain of CXCL12γ were analyzed against CXCL12α, 33 of them showed an overall good correlation (Fig. 1C), which suggests that CXCL12γ 1–68 domain and CXCL12α adopt identical tertiary structure. CXCL12γ 1–68 relaxation parameters (R1, R2 and ^15^N-^1^H NOes) were highly similar to those observed for monomeric CXCL12α [31] with the residues 10–64 being well structured. Residues 69–98 behaved differently: they were clustered between 8 and 8.5 ppm in the ^1^H dimension, suggesting they were poorly ordered in solution (Fig. 1D). According to TALOS, only a few residues are predicted to adopt an extended conformation (Fig. 1A). Seven N-H^N^ RDCs were observed in the γ extension between 2 and 7 Hz, presumably indicative of averaged RDCs due to important flexibility. This domain, with negative ^15^N-^1^H NOes and low R1 and R2 relaxation rates compared to the protein core, experienced fast timescale dynamics, confirming it was highly disordered in solution. Together, these data show that the C-terminal peptide is disordered and has no major effect on the structure of the first 68 residues of CXCL12γ.

The prevalence of such non structured protein segments, recently became increasingly recognized [32]. These domains, known as intrinsically disordered or natively unfolded, usually feature a unique combination of low overall hydrophobicity and high net charge, a point that clearly characterize the CXCL12γ C-terminal peptide. Proteins with such disordered regions are believed to performed critical functions, including molecular recognition through large and accessible interaction surfaces. In view of the highly basic nature of the CXCL12γ C-terminal domain, its disordered state, and the importance of GAG recognition for chemokine function, we then investigated the ability of CXCL12γ to interact with a variety of GAGs, including heparin (HP) HS, and dermatan sulfate (DS), and compared it to that of CXCL12α, β, which C-termini are distinct.

### CXCL12α, β and γ differently bind to GAGs

To determine the GAG binding ability of CXCL12α, β and γ isoforms we adopted a solid phase assay, in which reducing end biotinylated HP, HS or DS were captured on top of a streptavidin coated sensorchip, a system that mimics, to some extent, the cell membrane-anchored proteoglycans. Surface plasmon resonance (SPR) real time monitoring was exploited to measure changes in refractive index caused by the binding of chemokines to each of the immobilized GAGs.

**Figure 2:**
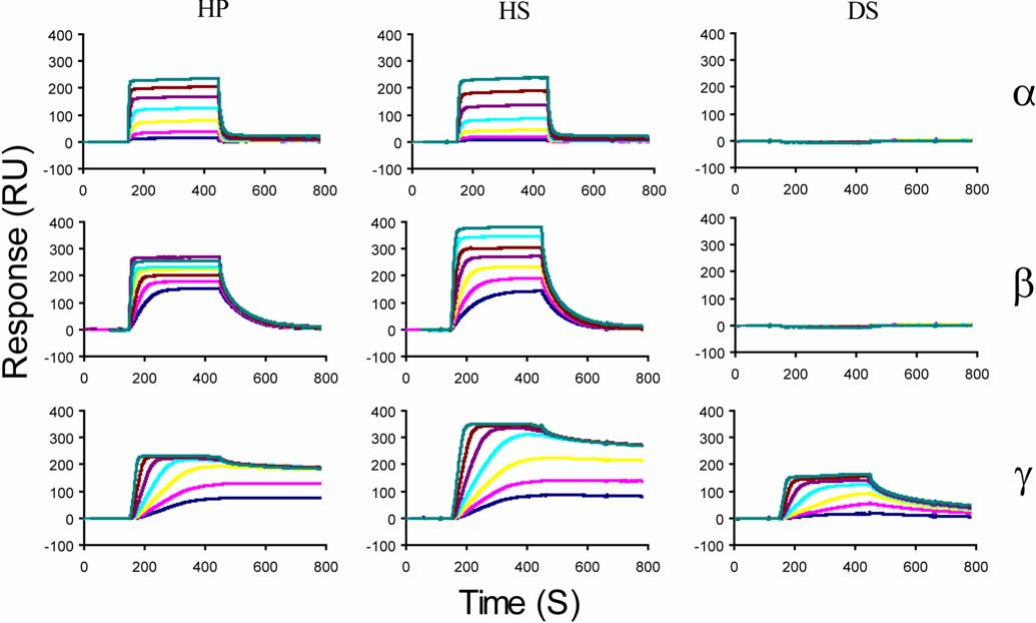
Analysis of CXCL12 binding to HP, HS and DS. SPR sensorgrams measured when CXCL12 were injected over HP, HS or DS activated sensorchips. The response in RU was recorded as a function of time for CXCL12α (26 to 300 nM), β (13 to 150 nM) and γ (2.6 to 30 nM).

Binding curves, obtained when the CXCL12 isoforms were flowed over the HP, HS and DS surfaces, showed marked differences (Fig. 2). These experiments first indicated that while CXCL12γ interacts with HP, HS and DS, CXCL12α and β only recognize HP and HS, suggesting that the C-terminal domain, which charaterizes the γ isoform, enables the chemokine to extend the range of GAGs to which it binds. Visual inspection of the sensorgrams also showed major differences during the dissociation phase. CXCL12α dissociated from the immobilized GAGs rapidly (binding curves returned to the base line within a minute), while CXCL12γ formed tight complexes, and CXCL12β displayed an intermediate behaviour. Preliminary analysis of the binding curves indicated that the binding rates were dominated by mass transfer, and global fitting of the binding curve returned values with low significances (see below). Because we generated data in which the association phase was allowed to proceed to equilibrium, affinity data were derived independently from the kinetic. By plotting R_eq_/C against R_eq_ for different concentrations of chemokine (in which R_eq_ are the steady state values at equilibrium and C the concentrations of injected chemokine), straight lines were obtained (data not shown) which slopes, corresponding to the equilibrium constant Kd, are reported in Table 1. These analyses demonstrated that CXCL12γ interacts with GAGs with an unprecedented propensity, featuring a 2 log increase compared to CXCL12α, and suggesting a strong participation of the C-terminal domain in the binding reaction.

**Table 1:** Equilibrium dissociation constant of CXCL12 for HP, HS and DS

	HP	HS	DS
CXCL12 α	93±6.1	200 nM±14	No binding
CXCL12 β	24.7 nM±2.6	53 nM±2.7	No binding
CXCL12 γ	0.91 nM±0.07	1.5 nM±0.2	4.8 nM±0.04

The equilibrium levels of bound CXCL12 were extracted from the sensorgrams of Fig. 2 at the end of the association phases (apart from the lowest CXCL12 concentrations, which in some cases did not reach equilibrium) and used to calculate the dissociation constant (Kd), using the Scatchard plot. Results are expressed in nM as means±SEM of 3 to 7 experiments

### Heparin derived oligosaccharides interact with CXCL12γ C-terminal domain and reduce its mobility

**Figure 3:**
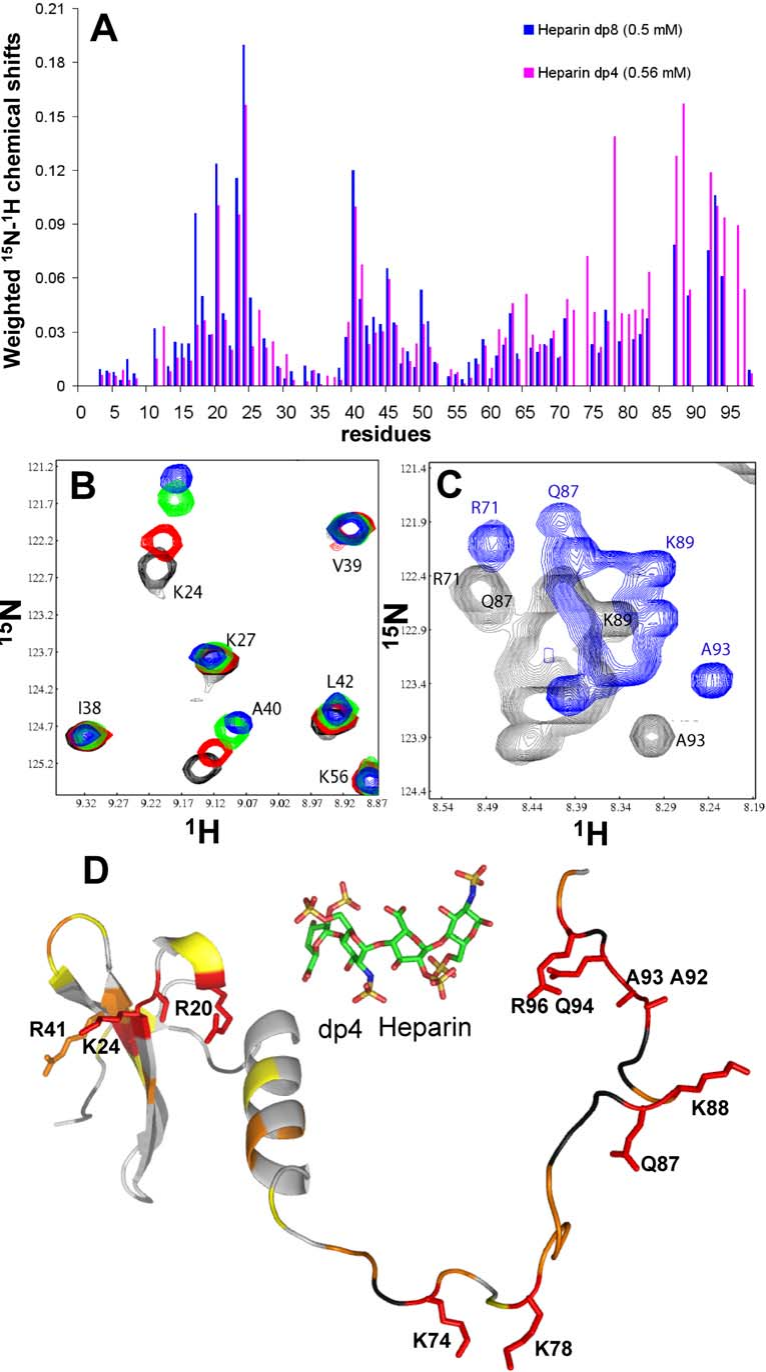
The interaction of CXCL12γ with dp4 and dp8 HP derived oligosaccharides reveals two main binding sites. (A) Weighted chemical shift differences √((ΔδH)^2^+(ΔδN/10)^2^) of CXCL12γ (0.2 mM) amide protons upon addition of dp4 (0.56 mM magenta) and dp8 (0.5 mM blue). Unassigned amide protons are left blank. Both the core structure and the C-terminus of CXCL12γ are affected upon oligosaccharide interaction. (B) ^15^N-HSQC in the 1-68 domain of CXCL12γ at 0 (black), 0.13 (red) 0.3 (green) and 0.5 mM (blue) dp4 concentration. (C) ^15^N-HSQC of CXCL12γ C-terminal residues at 0 (black) and 0.5 mM (blue) dp4 concentration. Highly overlapped C-terminal assignments could not be all followed upon interaction. It is nevertheless obvious that most residues are perturbed upon interaction with HP derived oligosaccharides. (D) Residues 69–98 of CXCL12γ where randomised by Simulating Annealing and manually attached to CXCL12α structure (PDB 1VMC). HP dp4 structure (extracted from PDB 1HPN) is also shown. Chemical shift variations upon dp4 addition are represented on CXCL12γ in yellow, orange, or red (respectively >0.03, 0.04 or 0.08 ppm) and dark grey (not determined). A continuous binding surface is formed on CXCL12γ core domain between R20 and R41 and the last 15 residues of the protein are highly affected by the interaction.

In view of the above data, which support the existence of additional GAG binding sites within the C-terminal domain of CXCL12γ, we performed titration experiments of ^15^N-CXCL12γ with different HP derived di-(dp2), tetra-(dp4) and octa-(dp8) saccharides. The CXCL12γ/oligosaccharide interactions were in fast exchange regime compared to NMR chemical shift timescale, typical of interactions in the µM-mM Kd range. Interaction with dp4 reached saturation, with an apparent Kd of about 250 µM. Several resonances in the γ extension were highly perturbed upon interaction (Fig. 3A). However, they could not be individually followed during titrations and backbone resonance assignment was performed on the ^15^N-^13^C-CXCL12γ/dp4 complex.

Interactions of dp2, dp4 and dp8 with CXCL12γ revealed two binding domains on the protein (Fig. 3). On the CXCL12γ core region, the most perturbed residues form a continuous surface, from R20 to R41 (Fig. 3D), including V23, K24, A40, and N45. This binding surface suggested an oligosaccharide orientation more or less perpendicular to the β sheet which differs from the previously described orientation of a dp12 in complex with a CXCL12α dimmer. In that case, the oligosaccharide also binds K24 and R41 but is aligned along the first β strand [24]. On the C-terminal extension, most of the residues were perturbed by the interaction in particular residues 83 to 97. Mab 6E9, which epitope consists of residues 78–80, still bound to the CXCL12γ/GAG complex (data not shown), further supporting the importance of the distal part of the C-terminus. Backbone chemical shifts from CXCL12γ/dp4 complex did not reveal any secondary structural changes compared to the free protein and no appearance of secondary structure elements in the C-terminal extension. ^15^N-^1^H heteronuclear NOes on the complex (data not shown) indicated however a significant decrease in mobility upon dp4 binding for the γ extension with positive NOe values for residues 82 to 89. A maximum NOe value around 0.2 for Q87 (data not shown) suggested nevertheless that, even in complex with HP derived oligosaccharides, the γ extension still exhibits important flexibility.

### The C-terminal domain and the binding sites in the core structure of CXCL12γ differently contribute to the binding

**Figure 4:**
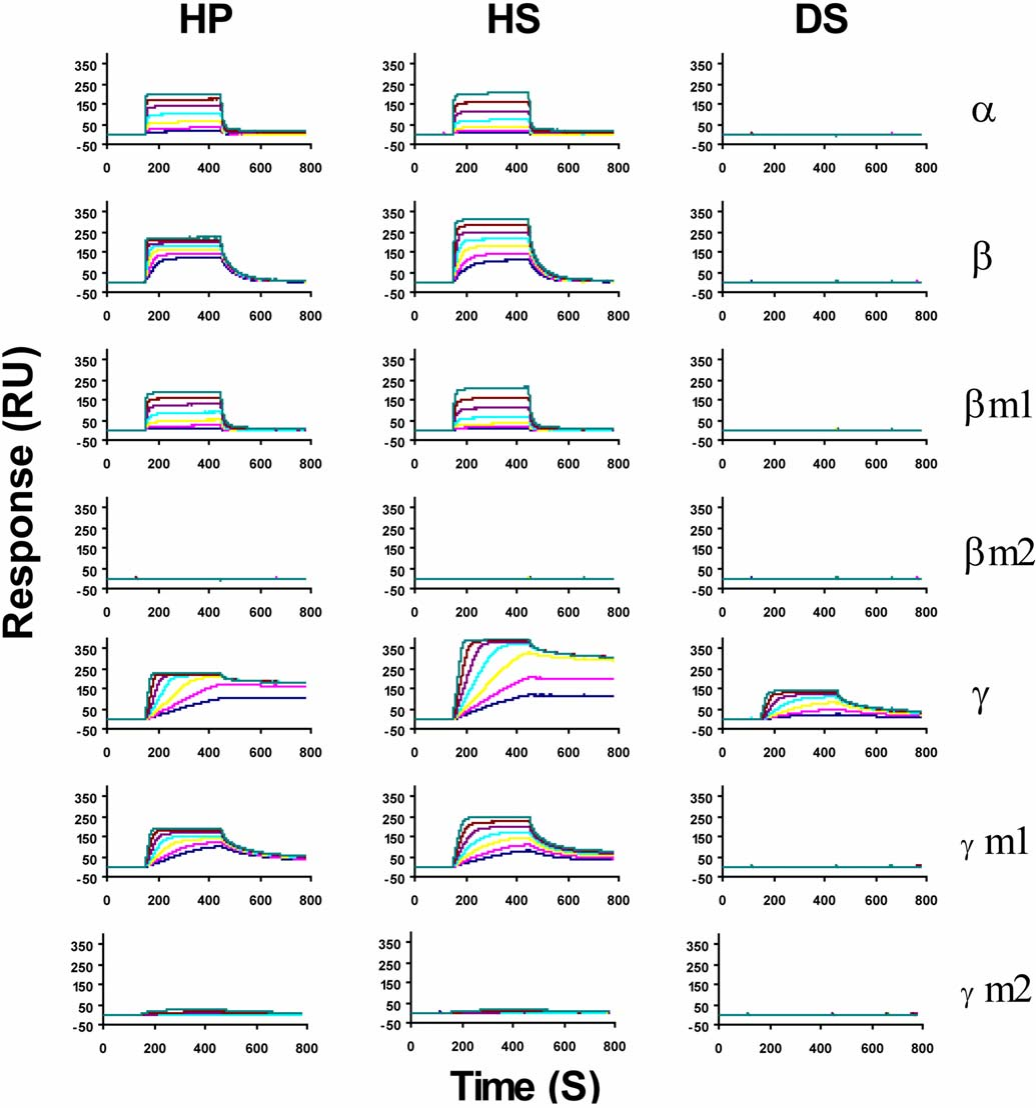
Analysis of wild type and mutant CXCL12 binding to immobilized GAGs. Binding of wild type and mutant CXCL12 were recorded as in Fig. 2. CXCL12α (26 to 300 nM), β, β-m1, β-m2 (13 to 150 nM), γ, γ-m1, γ-m2 (2.6 to 30 nM) were injected over GAG activated sensorchips and the response in RU was recorded as a function of time.

**Figure 5:**
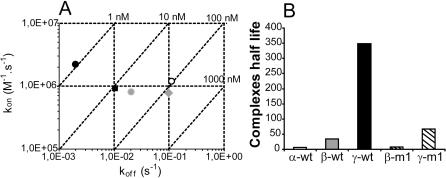
Association and dissociation rate constant of the CXCL12-GAG interaction. (A) Graphical summary of the data generated from the sensorgrams of Fig. 4, in which association (k_on_) and dissociation (k_off_) rate constants of CXCL12α (open circle), β (grey circle), β-m1 (grey square), γ (black circle) and γ-m1 (black square) for HP were determined as described. Differences were essentially observed along the k_off_ axis. (B) Dissociative half live of the different CXCL12/HP complexes.

To further analyze the respective GAG binding contributions of the core region and the C-terminal domain of CXCL12, mutations were introduced in both parts of the chemokine (see Fig. 1A) and their binding profiles were analyzed using the SPR assay (Fig. 4). As mentioned above, simultaneous fitting of the association and dissociation phases was not possible, presumably due to fast on rate which causes strong mass transport limitation during the association phase (data not shown), and possibly rapid rebinding of the dissociated molecules during the dissociation phase. The dissociation rates (k_off_) were thus measured at the beginning of the dissociation phase (where rebinding is limited because the number of free immobilized GAGs remains low) and the on rates (k_on_) were then calculated using the equilibrium dissociation constant (k_on_ = k_off_/Kd). Results are indicated in Fig. 5 and show that the C-terminal domain, while having limited effect on the on rate, essentially determines the velocity at which the formed complex dissociates. This is particularly marked for the γ isoform, which dissociates from HP with a k_off_ of 0.0019 M^−1^s^−1^ compared to 0.111 M^−1^s^−1^ for CXCL12α and 0.0204 M^−1^s^−1^ for CXCL12β. In agreement with these observations, mutations of the 3 basic residues present at the C-terminus of the β isoform (β-m1) did not change the on rate, but increased the off rate to a value of 0.098 M^−1^s^−1^, thus resulting in a behavior very close to that of CXCL12α (Fig. 5), with an overall affinity of 125 nM for HP and 192 for HS (compare with results in Table 1). Mutations in the core region (K24S/K27S), that completely abolished CXCL12α binding to HP/HS [24], were also introduced in β-m1 to yield a new mutant (β-m2; see Fig. 1). As expected, β-m2 did not bind anymore to GAGs.

Similarly, the effect on GAG binding of mutations introduced in the C-terminal domain of CXCL12γ was analyzed. Amongst the 18 basic residues of this domain, 9 were changed for Ser which removed the 3 typical HP binding clusters (Fig. 1A). Preliminary analysis performed with C-terminal synthetic peptides (residues 69–98) indicated that the wild type sequence required 0.88 M NaCl to be eluted from a HP affinity column, while the mutant peptide eluted at 0.28 M NaCl. This mutant peptide did not show any binding up to 200 nM using the SRP assay, demonstrating that these mutations very strongly decreased its binding capacity (data not shown). The GAG binding profile of the mutated full length chemokine (γ-m1, which includes these 9 mutations), was characterized. We observed that this mutant did not bind anymore to DS. This supports the view that the net charge of the CXCL12γ isoform C-terminal domain was involved in the broad GAG binding activity. As could have been anticipated, γ-m1 displayed an increased dissociation rate compared to the wild type chemokine (Fig. 5A), confirming the role of the C-terminal domain in the complex stability. The equilibrium dissociation constant for HP of this mutant was 10.4 nM (32 for HS). Thus, although this C-terminal domain by itself has a highly reduced binding capacity, the full length molecule still interacts quite strongly with HP and HS, suggesting a predominant role for the core domain. Consistently with this hypothesis, additional mutations in the core structure (γ-m2) dramatically decreased HP and HS binding, supporting further the critical importance of the core domain binding site for the interaction. CXCL12α/HP complex displayed an half live (ln[0.5]/k_off_) of 6 seconds, while CXCL12γ/HP complex was characterized by a half life of 350 seconds (Fig. 5B). Together, these data show that few key amino acids of the structured domain of CXCL12γ (in particular K24/27) constitute a strictly required binding site while, a number of positively charged residues of the unfolded C-terminus appears to primarily functions in stabilizing the formed complex.

Such different contributions between the two domains could be explained by the fact that electrostatic interactions are not always energetically positive. Favorable coulombic interactions formed in a final complex can be some times largely offset by the desolvation cost associated with the binding process [33], an effect that could occur within the unfolded and largely solvent accessible C-terminus of CXCL12γ. DNA-binding domains frequently have N-or C-terminal extensions, enriched in basic residues, and disordered in solution. The contribution of such basic tails, which increase the affinity for target DNA, has been studied in the context of protein-DNA interaction [34], but to our knowledge this has not yet been described for protein-GAG complex. In any case, the present findings support the view that for CXCL12γ, a large and unstructured C-terminal domain functions as an accessory “binding cassette” which, in cooperation with a restricted and well defined binding site in the core structure provides very tight binding to GAGs.

### CXCL12γ displays enhanced binding to cell surface expressed HS compared to CXCL12α

**Figure 6:**
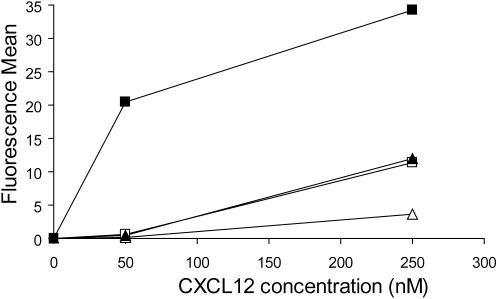
Flow cytometry analysis of CXCL12 interaction with cell surface GAGs. CHO-K1 parental cells (squares) or HS-deficient CHO-pgsD677 cells (triangles) were incubated with the indicated concentrations of CXCL12 α (open symbols) or γ (close symbols) and, after extensive washing to remove free chemokine, were labelled with K15C mAb and analyzed by flow cytometrey.

To investigate whether HS, in the context of the cell surface, also interacted more efficiently with CXCL12γ than with CXCL12α, we then compared the adsorption of these two isoforms on CXCR4 negative CHO cells by flow cytometry. The monoclonal antibody K15C, which recognize an epitope outside the HS binding site and present in all CXCL12 isoforms [35] were used for this purpose. Data are reported in Fig. 6, and show that binding to wild type CHO-K1 cells was greatly enhanced for CXCL12γ compared to CXCL12α. In particular, at low concentration (50 nM), CXCL12α did not displayed significant binding, while the γ isoform bound strongly to the cell surface, in agreement with the Biacore data (Fig. 2). These interactions were strongly reduced on HS deficient CHO-pgsD677 cells, demonstrating the importance of HS in the binding.

### CXCL12γ displays reduced binging to- and signaling through- CXCR4

**Figure 7:**
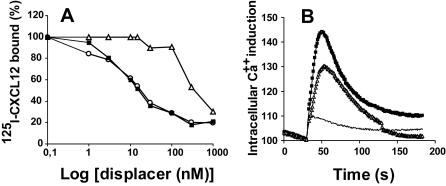
Comparative analysis of the CXCR4 binding and signaling properties of CXCL12α and γ. (A) ^125^I-CXCL12α (0.25 nM) was bound to CXCR4^+^ CEM cells in the presence of cold CXCL12α (squares), γ (triangle) or γ-m1 (circle). (B) Intracellular calcium mobilization induced by CXCL12α (squares), γ (triangles) isoforms or CXCL12α P2G (line) in A3.01 cells. CXCL12α P2G is a non signaling mutant of CXCL12α. Data are representative of three independent experiments.

To analyze the binding of CXCL12γ to CXCR4, we set up an assay, in which we compared the ability of the α and γ isoforms to compete with ^125^I-labeled CXCL12α. This was performed on T lymphoblastoid cell lines (CEM or A3.01), which do not express detectable amount of GAGs (data not shown), enabling the strict analysis of CXCL12/CXCR4 interaction. Results showed that CXCL12α and γ, although featuring identical receptor binding domain (localized in the N-terminus), behaved differently, the latter showing a reduced ability to bind to CXCR4, with an IC_50_ of 350 nM versus 15 nM for CXCL12α. This difference clearly relied on the C-terminal domain of CXCL12γ, since specific mutations within this domain (γ-m1) restored binding to a level comparable to that of CXCL12α (Fig. 7A). In agreement with this observation, CXCL12γ has a reduced ability to stimulate intracellular calcium mobilization compared to the α isoform (Fig. 7B).

The large amount of GAGs usually found at the cell surface, the reduced affinity of CXCL12γ for CXCR4 and its very high affinity for HS, suggest that within tissues the γ isoform might be predominantly in a bound form, associated to GAGs, and either stabilized to prevent proteolytic degradation and/or immobilized to allow continued and localized stimulation of cells.

### Conclusion

The binding of proteins to GAGs is the prerequisite for a large number of cellular processes and regulatory events. The chemokine system, in particular, strongly depends on HS, which are believed to ensure the correct positioning of chemokines within tissues.

In this report, we have shown that CXCL12γ, a new splice variant of CXCL12, displays an unusually high affinity for GAGs and investigated the structural determinants involved. The first 68 amino acids of the chemokine, common to all CXCL12 isoforms, comprised both the CXCR4 binding domain and a first, well defined, HS specific binding site. To this common platform is added, by alternative splicing of the *cxcl12* gene, different peptides which contain a second GAG binding domain, limited to 4 additional residues for CXCL12β but as long as 30 residues for CXCL12γ. This domain, which remains unfolded, appeared to mainly function by stabilizing the chemokine/HS complex. This, in combination with the structured first HS binding site, provides the protein with an unprecedented high affinity for HS. Interestingly, it has been described that polypeptide segments generated by alternative splicing are mostly intrinsically disordered [36]. This has been thought as a way to generate functional diversity without structural modification or complication. Our present findings fit well with this proposed mode of action. Thus, by encoding a singular domain, bearing the CXCR4 binding site, on which is added distinct C-terminus, CXCL12 may display distinct regulatory functions. The observation that the different CXCL12 isoforms mostly differ by their ability to interact with GAGs, offers an unprecedented opportunity to ascertain the importance of chemokine/GAG bindings in the regulation of *in vivo* cell migration. Regarding CXCL12γ, the remarkable conservation within mammals, of its entire C-terminal sequence is intriguing for a domain which presumably essentially triggers electrostatic interactions, and argues in favor of an important role played by this isoform. The observation that GAGs trigger a rapid and almost irreversible accumulation of CXCL12γ suggests that within tissues it should exist essentially in a bound form in nearby cells, presumably to allow continued and localized cellular stimulation. These data are compatible with a selective role of this isoform, and indicate that GAGs could be critical in orchestrating the CXCL12 mediated migration of cells, depending on the chemokine isoform and the nature of the GAGs to which it binds, either during development or post-natal life.

## Materials and Methods

### CXCL12 production and characterization

Murin *CXCL12γ* cDNA was inserted in a pET17b (Novagen) expression vector between NdeI and SpeI restriction sites, and checked by DNA sequencing. CXCL12γ was overexpressed overnight in *E. coli* BL21 (DE3) cells, with 0.4 mM IPTG, either in LB or M9 minimal medium supplemented with ^15^NH_4_Cl and ^12^C or ^13^C- glucose for isotopic enrichment. After 30 minutes of sonication at 4°C in 50 mM Tris pH 8.0 (buffer A), inclusion bodies were pelleted (20000g for 15 minutes) and washed with buffer A supplemented with 2M urea and 5 % Triton X100, then with 2 M urea and finally with buffer A. Inclusion bodies were solubilised for 15 min at 50°C in buffer A with 7.5 M GdCl_2_ and 100 mM DTT. Refolding was performed by rapid dilution with buffer A up to 1 M GdCl_2_. The mixture was gently stirred overnight at 4°C after addition of Complete protease inhibitors (Roche), then diluted 4 times with buffer A and loaded onto a 3 ml Source S column (Amersham) equilibrated in 20 mM Na_2_HPO_4_ pH 6.0. CXCL12γ was eluted with a NaCl gradient, concentrated and further purified on a G75 gel filtration column (Amersham) in 20 mM Na_2_HPO_4_, 150 mM NaCl pH 6.0. Purified material was analyzed by MALDI mass spectrometry and quantified by amino acids analysis. Wild type and mutants CXCL12α, β and γ were also produced by chemical synthesis, using the Merrifield solid-phase method and fluorenylmethyloxycarbonyl chemistry, as described [24].

### Preparation of heparin derived di- tetra- and octa-saccharides

Porcine mucosal HP was depolymerized with heparinase I. The digestion mixture was resolved from di-(dp2) to octa-(dp18) decasaccharide, and dp2 to dp8 were further purified by strong-anion-exchange HPLC as described [37].

### NMR experiments

NMR experiments were recorded at 30°C on Varian spectrometers (600 INOVA, 600 DD or 800 MHz with cryoprobe), processed with NMRpipe and analyzed with NMRview. CXCL12γ backbone assignment and relaxation experiments were recorded on 1 mM ^15^N-^13^C sample in 20 mM NaH_2_PO_4_ pH 5.7, 10% D_2_O, 0.01% NaN_3_ with protease inhibitors at 600 MHz. HNCACB, CBCA(CO)NH and HNCO, ^15^N-^1^H NOes and T_2_ experiments were from Varian Biopack and T_1_ experiment from [38]. Relaxation times were between 10 and 190 ms for T_2_ and 10 and 180 ms for T_1_. RDCs were measured as the difference between isotropic (25°C) and anisotropic (34°C) IPAP experiments [39] at 600 Mhz. 5% Bicelles (DMPC/DHPC 3:1 ratio) was used as the alignment medium with 180 µM of CXCL12γ in standard NMR buffer. The program MODULE was used to calculate the alignment tensor from the CXCL12α molecular shape and evaluate the correlations between experimental and backcalculated RDCs [40]. RDC data were evaluated against all CXCL12α published structures and fitted best 1VMC monomeric NMR structure [41]. Residues with the lowest correlations with respect to backcalculated data (11, 19, 20, 23, 35, 45, 46, 48 and 63) were excluded from the fit (and the calculation of the alignment tensor). These outliers are located mostly within regions of structural heterogeneity between the different published structures of CXCL12α. Titration with HP derived oligosaccharides was performed with 200 µM ^15^N-CXCL12γ in the NMR buffer.

### Surface plasmon resonance based binding assay

Size defined HP (6 kDa), HS and DS were biotinylated at their reducing end, and immobilized on a Biacore sensorchip. For this purpose, flow cells of a CM4 sensorchip were functionalized with 2500 to 2800 resonance units (RU) of streptavidin as described [24] and biotinylated HP (5 µg/ml), HS (25 µg/ml) and DS (15 µg/ml) in HBS (10 mM HEPES, 150 mM NaCl, 3 mM EDTA, 0.005% surfactant P20, pH 7.4) were injected across the different flow cells to obtain immobilization levels of 40, 70 and 140 RU respectively. One flow cell was left untreated and served as negative control. For binding assays, 250 µl of CXCL12 were simultaneously injected, at a flow rate of 50 µl/min, over the control and the different GAG surfaces, after which the formed complexes were washed with running buffer for 5 min. The sensorchip surface was regenerated with a 3 minute pulse of 2 M NaCl. Control sensorgrams were subtracted on line from GAG sensorgrams, and results analyzed using the Biaeval 3.1 software.

### Binding of CXCL12 to CXCR4 and cell surface HS

CEM cells (10^7^ cells/ml) were incubated with 0.25 nM of ^125^I-CXCL12α (Perkin-Elmer, 2200 Ci/mmol) and a range of concentrations of unlabelled CXCL12 (α, γ or γ-m1) in 100 µl of PBS for 1h at 4°C. Incubations were stopped by centrifugation at 4°C. Cell pellets were washed twice in ice-cold PBS, and the associated radioactivity was counted. For measuring the ability of CXCL12 to interact with cellular HS, the CXCR4 negative CHO-K1 or HS-deficient CHO-pgsD677 (ATCC) were incubated with the ckemokine and after removal of unbound proteins, were labelled with an anti-CXCL12 mAb (clone K15C) and a PE-conjugated secondary antibody. Immunolabelled cells were analysed by flow cytometry using a FacsCalibur (BD Biosciences).

### Intracellular calcium release responses

Intracellular calcium measured in CXCR4-expressing cells loaded with fluo-4-AM (Interchim) was conducted in a Mithras LB 940 counter (Berthold Technologies). Briefly, A3.01 cells were incubated for 45 min at 37°C in the load buffer (10 mM Hepes, 137.5 mM NaCl, 1.25 mM CaCl_2_, 1.25 mM MgCl_2_, 0.4 mM NaH_2_PO_4_, 1 mM KCl, 1 mM Glucose) with 0.1% of pluronic acid and 0.5 mM of Fluo4-AM (10^6^ cells/mL). After a washing step, cells were suspended in load buffer at a final concentration of 2×10^6^ cells/mL and stored at 4°C. For intracellular calcium measurements, aliquots of cells (2×10^5^ cells) were preincubated at 37°C for 1 min, then placed in a 96-well flat bottom plate. Fluorescence emission was recorded at 535 nM (excitation at 485 nM) every second before (basal fluorescence) and after programmed injection of different concentration of the ligands. Maximum and minimum fluorescence values were determined after addition of Triton X-100 and EDTA, respectively. Data are expressed as fluorescence increment rate after ligand addition.
